# Abbreviated dual antiplatelet therapy after percutaneous coronary intervention with drug coated balloons in acute coronary syndromes: insights from the SWEDEHEART registry

**DOI:** 10.1093/ehjcvp/pvag032

**Published:** 2026-05-07

**Authors:** Anton Håkansson, Sacharias von Koch, Axel Dahlgren, Christian Reitan, Sasha Koul, Stefan James, Tomas Jernberg, Per Grimfjärd, Elmir Omerovic, Oskar Angerås, David Erlinge, Moman A Mohammad

**Affiliations:** Division of Cardiology, Department of Clinical Sciences, Lund University, BMC I12, 221 84 Lund, Sweden; Division of Cardiology, Department of Clinical Sciences, Lund University, BMC I12, 221 84 Lund, Sweden; Division of Cardiology, Department of Clinical Sciences, Lund University, BMC I12, 221 84 Lund, Sweden; Department of Clinical Sciences, Cardiology, Karolinska Institutet, Danderyd Hospital, 182 88 Stockholm, Sweden; Division of Cardiology, Department of Clinical Sciences, Lund University, BMC I12, 221 84 Lund, Sweden; Deptartment of Medical Sciences, Uppsala University Hospital, 751 85 Uppsala, Sweden; Department of Clinical Sciences, Danderyd Hospital, Karolinska Institutet, 182 88 Stockholm, Sweden; Department of Medical Sciences, Västmanland hospital, Västerås, 721 89 Västerås, Sweden; Division of Cardiology, Department of Molecular and Clinical Medicine, Sahlgrenska Academy, University of Gothenburg, 413 45 Gothenburg, Sweden; Department of Thoracic Surgery and Cardiology, Sahlgrenska University Hospital, 413 45 Gothenburg, Sweden; Institute of Medicine, Sahlgrenska Academy, University of Gothenburg, 413 90 Gothenburg, Sweden; Division of Cardiology, Department of Clinical Sciences, Lund University, BMC I12, 221 84 Lund, Sweden; Division of Cardiology, Department of Clinical Sciences, Lund University, BMC I12, 221 84 Lund, Sweden

**Keywords:** Acute coronary syndrome, Dual antiplatelet therapy, Drug-coated balloon

## Abstract

**Aims:**

The absence of stent implantation when using drug-coated balloons (DCBs) may decrease the required duration of dual antiplatelet therapy (DAPT). In the light of this, this study aimed to evaluate outcomes for patients with acute coronary syndromes (ACSs) treated with abbreviated vs. standard DAPT after DCB-only percutaneous coronary intervention (PCI).

**Methods and results:**

Patients enrolled in the SWEDEHEART registry between June 2013 and February 2022, treated exclusively with DCBs for ACS, were included. Only patients discharged with ticagrelor as the P2Y12-receptor inhibitor were included. Patients were categorized by intended DAPT duration at discharge. The primary outcome was net adverse clinical events (NACE) at 1 year from discharge date, defined as the first occurrence of all-cause death, stroke, myocardial infarction, or major bleeding. The primary analysis used inverse-probability-of-treatment-weighted Cox regression. Among 1128 patients (141 abbreviated DAPT, 986 standard DAPT), NACE occurred in 25 patients (crude 17.7%; weighted 17.8%) in the abbreviated-DAPT arm and 133 patients (crude 13.5%; weighted 13.8%) in the standard-DAPT arm, corresponding to a weighted hazard ratio of 1.29 (95% confidence interval 0.81–2.03; *P* = 0.28). Results were consistent across pre-specified sensitivity analyses. Due to the small sample size, variance was generally high.

**Conclusion:**

In this nationwide registry-based analysis, abbreviated DAPT following DCB-only PCI in ACS was not associated with a statistically significant difference in NACE. However, the confidence intervals were wide and did not exclude clinically meaningful harm. The findings should be regarded as hypothesis-generating and indicate the need for more comprehensive evidence before abbreviated DAPT is routinely adopted in this setting.

## Graphical Abstract

**Graphical Abstract:**
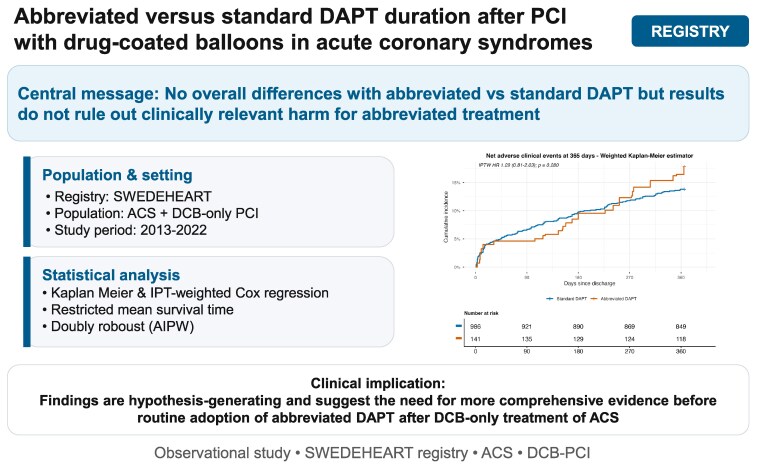

## Introduction

Drug-coated balloons (DCBs) are increasingly used in contemporary percutaneous coronary intervention (PCI). Especially in the setting of in-stent restenosis (ISR) and small vessel disease, but also in wider populations, such as for larger *de novo* lesions.^1–4^ Drug-coated balloons leave no permanent scaffold, reducing thrombogenicity and potentially allowing shorter dual antiplatelet therapy (DAPT).^5^ With the growing use of DCB, the optimal antiplatelet therapy following DCB–PCI has become a subject of debate, particularly in patients with acute coronary syndrome (ACS) who are at higher risk of ischaemic events.

Albeit the optimal regimen remaining unclear, de-escalated or abbreviated DAPT has been shown to be a viable alternative for ACS patients undergoing PCI with drug eluting stents in several trials and meta-analyses in recent years.^6–10^ It results in a decreased bleeding risk, often without a clinically significant increase of the ischaemic risk. Because of this, various types of de-escalated, or abbreviated, regimens are being increasingly used, especially for high bleeding risk patients. Current European Society of Cardiology and corresponding American guidelines recommend 12 months of DAPT as the default strategy for ACS patients with the option to de-escalate-, or abbreviate DAPT the regimens if clinically motivated (i.e. by high bleeding risk).^9,11^ However, these recommendations are almost exclusively based on trials involving stent implantation, which remains the standard of care for ACS patients. Consequently, the extrapolation of the guidelines to DCB-settings remains an issue. Several expert consensus-documents have proposed de-escalated DAPT as a possible standard for post-DCB care but the lack of studies on the topic has meant there is insufficient rigorous data to guide clinical decision-making.^1,12^

The trial REC-CAGEFREE II found abbreviated ticagrelor-based DAPT non-inferior to standard therapy.^13^ However, it remains the only large study exploring this topic and while other DCB-trials have also reported DAPT durations, they do not directly compare different DAPT strategies within the DCB population.^3,14–17^

To address these knowledge gaps, we conducted a nationwide cohort study using data from the Swedish Web-System for Enhancement and Development of Evidence-Based Care in Heart Disease Evaluated According to Recommended Therapies (SWEDEHEART) registry. We aimed to assess outcomes for ACS patients receiving abbreviated ticagrelor-based DAPT after DCB-only PCI, hypothesizing that it might be viable treatment option for these patients.

## Methods

### Design

**Figure 1:**
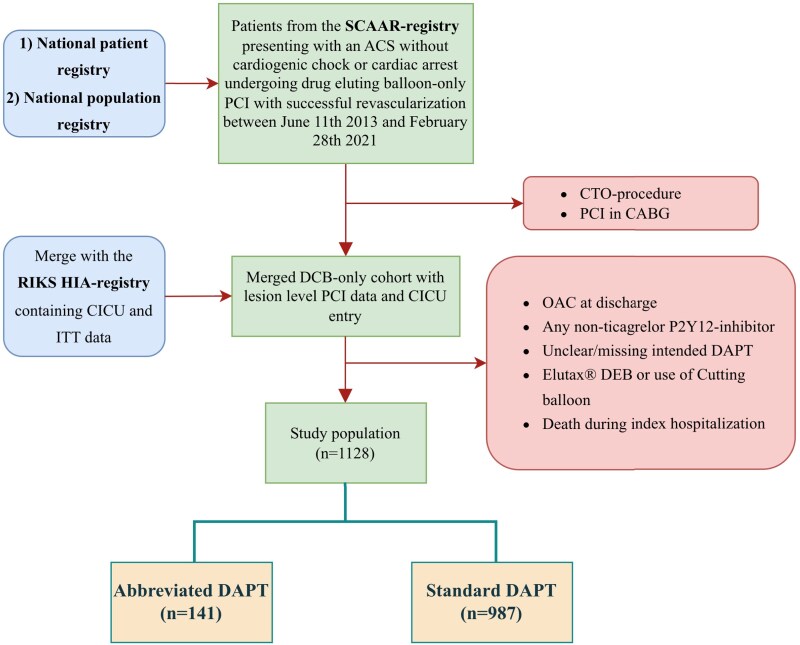
Flow-chart of study population selection. Exclusion and inclusion criteria. Flowchart of data management including data sources, patient inclusion, and crude matching process. Shown are the numbers of patients remaining after each step of the inclusion and exclusion criteria. ACS, acute coronary syndrome; DCB, drug coated balloon; PCI, percutaneous coronary intervention; CABG, coronary artery bypass graft; DAPT, dual antiplatelet therapy; SWEDEHEART, Swedish Web-System for Enhancement and Development of Evidence-Based Care in Heart Disease Evaluated According to Recommended Therapies.

This was an observational longitudinal cohort study based on data from the SWEDEHEART registry, a nationwide cardiac registry. It adheres to the Strengthening the Reporting of Observational Studies in Epidemiology (STROBE) guidelines for observational studies and was approved by Swedish Ethical Review Authority (Dnr 2023-00201-01). All adult patients in the SWEDEHEART registry hospitalized with ACS, defined as ST-elevation myocardial infarction, non-ST-elevation myocardial infarction, or unstable angina, between 11 June 2013, and 28 February 2022, were identified. Patients who underwent PCI with DCB angioplasty as the only revascularization strategy during the index hospitalization were included in the study. If bailout stenting was used, the patient was excluded. Patients with incomplete revascularization, oral anticoagulation, any PCI in a chronic total occlusion or coronary artery bypass graft, any non-ticagrelor-P2Y12-inhibition as well as any abbreviated duration of acetylsalicylic acid were excluded, *Figure 1*. As introduction of a patients’ intended treatment duration into the registry is part of discharge procedure, only patients who survived until discharge were eligible for inclusion. Patients were stratified according to the intended strategy of DAPT at discharge: a ‘any abbreviated’ DAPT group (≤6 months ticagrelor) and a standard DAPT group (≥12 months).

### Time-to-event and outcomes

The date of discharge was used as time zero for all endpoints and the patients were then followed prospectively within the registry for 365 days. The primary outcome was net adverse clinical events (NACE) at 1 year defined as the first occurrence of: (i) all-cause death, (ii) myocardial infarction (MI), (iii) ischaemic stroke, or (iv) clinically significant bleeding event. All outcomes are defined in detail in Supplementary material online, *Table S1*. Death status was ascertained by linking the National Board of Health and Welfare to the National Population Register. Dates and rates of stroke and bleeding outcomes were obtained through the National Patient Registry. The *ICD* codes used to define bleeding were chosen to reflect bleeding events corresponding to Bleeding Academic Research Consortium Type 2, three-events and included haemorrhagic stroke.^18^ Fatal bleeding was included in the all-cause death endpoint. To ensure that only clinically relevant bleeding events were used, only bleeding diagnoses linked to hospital inpatient care were included in the analysis. The sensitivity of fatal bleeding or bleeding requiring hospitalization in Swedish registries has been previously validated and is 99.5% and 84.5%.^19^ Information on MI was obtained from SWEDEHEART registry and defined as a subsequent entry to a coronary care unit with a discharge diagnosis of MI according to the fourth universal definition of MI.^20^ All outcomes were ascertained up to 1 year after discharge. No data on the true, per protocol regimen was available. Overall adherence to the intention to treat (ITT) regimen in other Swedish observational ACS cohorts has been shown to be around 80%, with 5% P2Y12-inhibitor-crossover.^21^

### Statistical analysis

Cumulative incidence of the primary and secondary endpoints was estimated using inverse-probability-of-treatment-weighted (IPTW) Kaplan–Meier estimators where death was a component of the endpoint and using the Aalen–Johansen estimator with death as a competing risk otherwise. The target estimate was the average treatment effect (ATE) of abbreviated vs. standard DAPT on 365-day NACE. Propensity scores were estimated using logistic regression with planned DAPT duration (ITT) as the dependent variable. Covariate selection followed the principles suggested by Hernán and Robbins, including common causes of treatment and/or outcome while excluding variables with instrument-like behaviour. That is, strong predictors of treatment assignment without independent prognostic value after conditioning on measured covariates.^22,23^ Calendar year was excluded on this basis after empirical confirmation on instrument-like behaviour [adjusted Cox hazard ratio (HR) per year 1.02, *P* = 0.61].^23^ Other variables that were screened and not included are shown in Supplementary material online, *Table S2*. The primary IPTW set was restricted to 15 variables to avoid over-adjusting.^24,25^

The primary analysis used IPTW with stabilized ATE weights applied to Cox proportional hazards models for each outcome, with robust (sandwich) standard errors. For the primary analysis crude weights were used. Analyses using weights truncated at first and 99th percentile and overlap weights were also performed to estimate output dependence on extreme weights.^26^ Covariate balance before and after weighting was evaluated using absolute standardized mean differences (SMDs). The proportional hazards assumption was tested in the primary analysis for increased interpretability of produced HRs.

Five sensitivity analyses were performed. First, a slim adjusted Cox outcome-model including five covariates selected as the strongest shared confounders across endpoints (previous bleeding, age, estimated glomerular filtration rate (eGFR), culprit lesion type, and ACS presentation type), constrained by the events-per-variable ratio. Second, alternative IPTW models were created, Supplementary material online, *Table S2*. Third, average treatment effect in the overlap-weights (ATO), targeting the population in clinical equipoise, were calculated in order to address potential positivity violations.^27^ Fourth, augmented inverse probability weighting (AIPW) with SuperLearner (logistic regression, penalized logistic regression and random forest) estimation and five-fold cross-fitting was performed. Outcomes from this analysis report risk differences and risk ratios for binary 365-day outcomes as a doubly robust complement to the primary IPTW analysis. Fifth, we estimated the IPW-adjusted difference in restricted mean survival time (RMST) at τ = 365 days by integrating the IPTW-weighted Kaplan–Meier curves and computing the area between groups, following the method of Conner *et al*.^28^ The model provides an outcome measure that does not assume proportional hazards. Note: Survival being defined as not having had any NACE, not time until death. Lastly, a pre-specified subgroup analysis estimated within-stratum treatment effects as well as the corresponding treatment × culprit lesion type-interaction term.

As detailed in the supplement, a Holm–Bonferroni analysis plan was created to adjust for multiplicity. Data management and statistical analysis was performed in Stata version 19.0 and 19.5^29^ and R version 4.4.2.^30^

## Results

### Baseline characteristics

A total of 1128 patients treated with DCB-only PCI for ACS were included in the analysis, *Table 1*, *Figure 1*. Of these, 141 patients (12.5%) had an intended short DAPT duration (≤6 months) and 987 patients (87.5%) had a standard ≥12-month intended duration. The overall ischaemic risk in the cohort was high, with a high prevalence of previous PCI and MI. Based on the PRECISE-DAPT-score, there were differences in baseline bleeding risk between populations, *Table 1*. The mean DCB of deployed DCB was 2.7 mm, indicating mainly small-vessel deployment, Supplementary material online, *Table S3*. Further detailed procedural data is shown in Supplementary material online, *Table S3*.

**Table 1:** Baseline characteristics of the study population

Characteristics	Overall (*N* = 1 128)^a^	Standard DAPT (*N* = 987)^a^	Abbreviated DAPT (*N* = 141)^a^	SMD^b^
Age, years	65.8 (11.2)	65.7 (11.1)	66.5 (11.8)	−0.07
Sex	795 (70.5%)	710 (71.9%)	85 (60.3%)	0.25
BMI, kg/m^2^	27.7 (4.5)	27.8 (4.6)	27.2 (4.4)	0.13
Smoking status				0.25
Never smoked	446 (39.5%)	386 (39.1%)	60 (42.6%)	
Ex-smoker >1 month	456 (40.4%)	408 (41.3%)	48 (34.0%)	
Current smoker	201 (17.8%)	176 (17.8%)	25 (17.7%)	
Unknown	25 (2.2%)	17 (1.7%)	8 (5.7%)	
Type of ACS				0.39
STEMI	205 (18.2%)	194 (19.7%)	11 (7.8%)	
Unstable angina	332 (29.4%)	276 (28.0%)	56 (39.7%)	
NSTEMI	591 (52.4%)	517 (52.4%)	74 (52.5%)	
PRECISE-DAPT ≥25	265 (23.5%)	218 (22.1%)	47 (33.3%)	0.25
In-stent culprit lesion	489 (43.4%)	432 (43.8%)	57 (40.4%)	0.07
Diabetes mellitus	333 (29.5%)	289 (29.3%)	44 (31.2%)	0.04
Hypertension	568 (50.4%)	491 (49.7%)	77 (54.6%)	0.10
Previous myocardial infarction	522 (46.3%)	456 (46.2%)	66 (46.8%)	0.01
Heart failure	102 (9.0%)	84 (8.5%)	18 (12.8%)	0.14
Previous PCI	689 (61.1%)	606 (61.4%)	83 (58.9%)	0.05
Previous CABG	59 (5.2%)	52 (5.3%)	7 (5.0%)	0.01
Peripheral artery disease	53 (4.7%)	43 (4.4%)	10 (7.1%)	0.12
Significant bleeding within 1 year before admission	52 (4.6%)	41 (4.2%)	11 (7.8%)	0.15
Chronic kidney disease	53 (4.7%)	44 (4.5%)	9 (6.4%)	0.09
COPD	83 (7.4%)	71 (7.2%)	12 (8.5%)	0.05
eGFR (MDRD), mL/min/1.73 m^2^	84.8 (26.9)	85.5 (26.3)	80.3 (30.5)	0.18
Haemoglobin, g/L	138.7 (15.8)	139.1 (15.5)	136.4 (17.3)	0.16
ACE inhibitor at discharge	598 (53.0%)	530 (53.7%)	68 (48.2%)	0.11
ARB at discharge	326 (29.0%)	286 (29.0%)	40 (28.6%)	0.01
Beta-blocker at discharge	934 (82.8%)	817 (82.8%)	117 (83.0%)	0.01
Statin at discharge	1084 (96.2%)	949 (96.2%)	135 (95.7%)	0.03
Diuretic at discharge	194 (17.2%)	168 (17.0%)	26 (18.4%)	0.04
Intended ticagrelor duration				0.68
1 month	23 (2.0%)	0 (0.0%)	23 (16.3%)	
3 months	48 (4.3%)	0 (0.0%)	48 (34.0%)	
6 months	70 (6.2%)	0 (0.0%)	70 (49.6%)	
≥12 months	987 (87.5%)	987 (100.0%)	0 (0.0%)	

Baseline demographic and medical characteristics for the standard DAPT group and the abbreviated DAPT group. Differences between groups were calculated using standardized mean differences.

BMI, body mass index; DAPT, dual antiplatelet therapy; ACS, acute coronary syndrome; STEMI, ST-elevation myocardial infarction; NSTEMI, non-ST-elevation myocardial infarction; PCI, percutaneous coronary intervention; CABG, coronary artery bypass graft; COPD, chronic obstructive pulmonary disease; ACE, angiotensin converting enzyme; ARB, angiotensin receptor blocker; eGFR, estimated glomerular filtration rate; PRECISE-DAPT, PREdicting bleeding Complications In patients undergoing Stent implantation and subsEquent Dual Anti Platelet Therapy.

^a^Number of patients in unweighted population.

^b^Standardized mean difference.

### Model diagnostics and assumptions

A density plot of propensity scores by treatment arm are displayed in Supplementary material online, *Figure S1*. Two variables had a SMD greater than the threshold of 0.1 at baseline (hospital frailty risk score and ACS type).^31^ After IPT weighting no adjustment-variable differed significantly between populations, Supplementary material online, *Figure S2*. Detailed data on weight diagnostics and the frailty risk score are provided in Supplementary material online, *Tables S4* and *S5*. Test of proportional hazards in the primary analysis indicated moderate, but statistically significant, non-proportionality, *P* = 0.03. Plot of scaled Schoenfeld residuals are shown in Supplementary material online, *Figure S3*.

### Primary analysis

The primary composite endpoint of NACE at 365 days occurred in 25 patients (17.7%) in the abbreviated-DAPT arm and 133 patients (13.5%) in the standard-DAPT arm, corresponding to IPTW-weighted cumulative incidence of 17.8% and 13.8%, respectively, *Table 2*.

**Table 2:** Inverse probability of treatment weighted Cox regression for primary and secondary endpoints

Outcome	Abbreviated DAPT (*n* = 141)		Standard DAPT (*n* = 986)		
	Events, *n* (%)	Weighted %	Events, *n* (%)	Weighted %	IPTW HR (95% CI); *P*
**Net adverse clinical events**	25 (17.7)	17.8	133 (13.5)	13.8	1.29 (0.81–2.03); 0.28
**Secondary endpoints**					
**All-cause death**	6 (4.3)	3.1	20 (2.0)	2.2	1.41 (0.54–3.66); 0.48
**Myocardial infarction**	17 (12.1)	11.4	88 (9.0)	9.1	1.24 (0.71–2.15); 0.45
**Stroke**	1 (0.7)	0.8	6 (0.6)	0.7	1.11 (0.13–9.17); 0.93
**Major bleeding**	8 (5.8)	6.2	41 (4.2)	4.3	1.45 (0.62–3.36); 0.39

Abbreviated DAPT defined as ≤6 months of DAPT; standard DAPT as ≥12 months. Crude percentages reflect unadjusted event proportions; weighted percentages are derived from stabilized IPTW (ATE) weights based on the primary IPTW model. Hazard ratios from IPTW-weighted Cox proportional hazards regression with robust standard errors.

DAPT, dual antiplatelet therapy; HR, hazard ratio.

**Figure 2:**
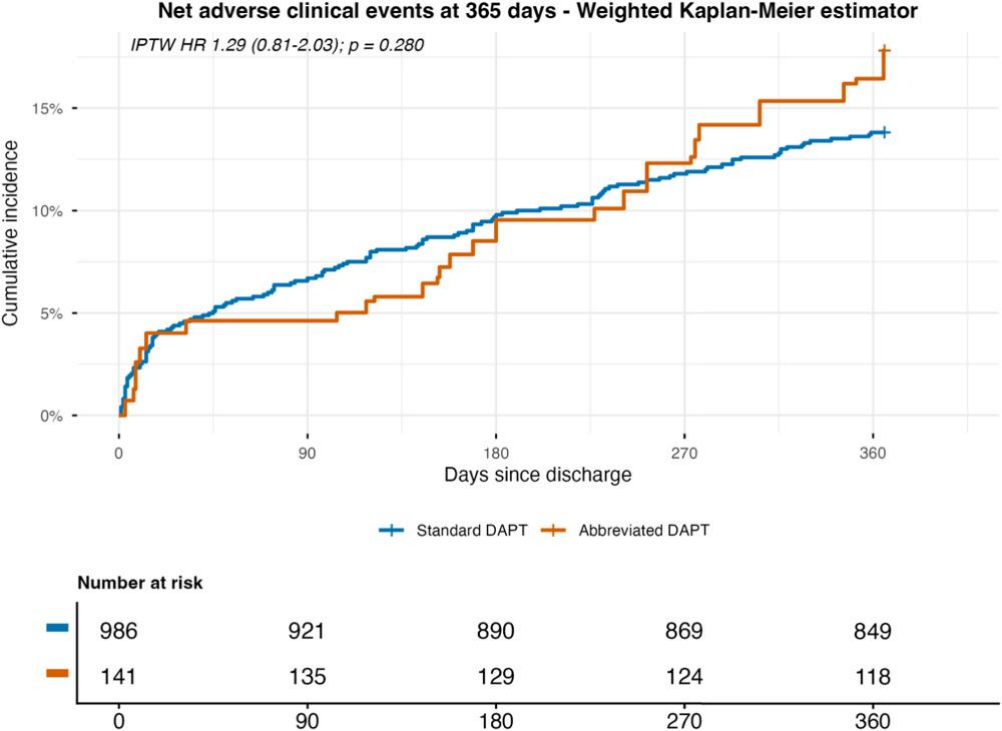
Weighted Kaplan–Meier graphs for the primary outcome net adverse clinical events. The figure shows the graph of the weighted Kaplan–Meier cumulative incidence for the primary endpoint, net adverse clinical events at 1 year after discharge. Provided statistical test is the weighted Cox regression hazard ratio for the same endpoint. The table representing the number of patients at risk represent the number of patients left in the risk-model that are still contributing weights.

After IPTW, abbreviated DAPT was not associated with any statistically significant difference in the 1-year hazard of NACE [HR 1.29, 95% confidence interval (CI) 0.81–2.03; *P* = 0.28], *Table 2* and *Figure 2*. The *E*-value for shifting the HR to 1.00 in this analysis was 1.67, indicating a low threshold for unmeasured confounding significantly affecting results.^32^

### Secondary analyses

**Figure 3:**
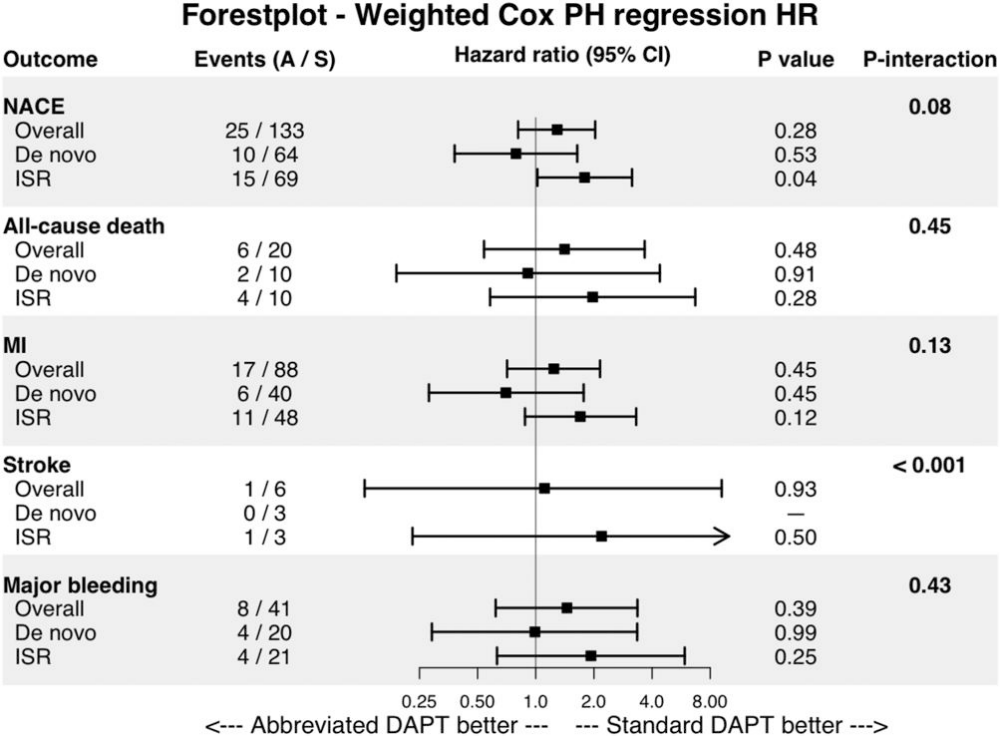
Forest plot showing sub-group-specific outcomes for primary endpoint. Forest plot point estimates and corresponding confidence interval for the weighted hazard ratio for net adverse clinical events comparing abbreviated vs. standard dual antiplatelet therapy. Provided statistical test is the weighted Cox regression hazard ratio for the primary endpoint (net adverse clinical events). Results indicated by ‘overall’ correspond to the primary, unstratified cohort. Results in the ‘De novo’ and ‘ISR’ rows are based on stratified weighted cox regressions, without re-weighting in specific subgroup of culprit lesion. Interaction *P*-values were estimated in the overall population. PH, proportional hazards; HR, hazard ratio; NACE, net adverse clinical event, MI, myocardial infarction; DAPT, dual antiplatelet therapy.

For secondary endpoints, unweighted Kaplan–Meier, IPTW-weighted Kaplan–Meier, and the IPTW-weighted Aalen–Johansen estimator all showed similar results, Supplementary material online, *Table S6*. The weighted HRs were above one for all secondary endpoints. However, since counts were generally low, there was limited statistical precision for these findings. *Table 2* and *Figure 3*. Hospitalization due to intestinal bleeding was the most common bleeding event, Supplementary material online, *Table S7*.

### Sensitivity analyses

The primary finding was robust across all pre-specified sensitivity analyses, Supplementary material online, *Table S8*. Applying trimmed weights yielded a NACE HR of 1.34 (95% CI 0.86–2.09), and overlap-weighted (ATO) analysis produced an attenuated but directionally consistent estimate (HR 1.17, 95% CI 0.75–1.80). The adjusted Cox model as well as alternative IPTW models also provided consistent results, Supplementary material online, *Tables S8* and *S9*.

The doubly-robust AIPW estimator yielded a risk difference of +5.6 percentage points (95% CI −2.9 to 14.1; *P* = 0.20) and a risk ratio of 1.40 (95% CI 0.90–2.20; *P* = 0.14) at 365 days, consistent in direction with the IPTW-Cox HR. Inverse-probability-of-treatment-weighted-weighted restricted mean survival time analysis at *τ* = 365 days showed a ΔRMST of +0.72 days in favour of abbreviated DAPT.

### Subgroup analysis

Among patients with *de novo* lesions as culprit, there was no difference between treatment arms. In contrast, there was a possible sign of treatment harm in patients with in-stent culprit lesions (HR 1.79; 95% CI 1.02–3.15; *P* = 0.043). However, there was no statistically significant interaction effect for lesion type, *Figure 3*.

## Discussion

### Key findings

Abbreviated DAPT was not associated with a statistically significant increase in NACE compared with standard DAPT after ACS treated with only DCBs. However, point estimates across both primary and exploratory endpoints favoured standard DAPT, and confidence intervals were wide, leaving clinically relevant treatment harm possible.

### Interpretation

Although nationwide, the cohort represents a sub-group of ACS patients clinically indicated for DCB–PCI based on contemporary evidence and does not represent a complete sample of the ACS population. The proportion of previous MI and PCI is high, and the cumulative incidence of all-cause death during the first year of follow-up is low compared with other contemporary Scandinavian ACS-cohorts.^33^ Compared with the only randomized data on the topic (the REC-CAGEFREE II trial), our population exhibits a markedly higher proportion of patients with MI and in-stent culprit lesions at presentation.^13^ The effects of this are possibly mirrored by that the cumulative incidence of the primary endpoint is almost double that reported in the intervention arm of the REC-CAGEFREE II trial (even though our endpoint does not include planned revascularization or out-patient bleeding events).^13^ Summarily, this suggests that the adoption of DCB in the ACS setting in Sweden differs from the population in the only randomized data published in support of abbreviated DAPT after DCB-only PCI after ACS to date.

Despite the evident differences between the populations, our primary results do not contradict the findings in the REC-CAGEFREE II-trial, which demonstrated that abbreviated ticagrelor-based DAPT after DCB-treated ACS was non-inferior to a 12-month regimen at 1 year.^13^ Our results showed no statistically significant difference between treated groups across an extensive set of sensitivity analyses. As the interpretation of the primary analyses is complicated by the violated proportional hazards assumption, the completely neutral RMST results should be specifically mentioned in this context. Nonetheless, all our findings should be viewed in light of the small sample size and high variance. The confidence intervals include significantly elevated HRs and potentially clinically relevant treatment harm.

This possible harm might be reflected by the increased HR for NACE among patients presenting with a culprit in-stent lesion in this cohort. In-stent restenosis-lesions exhibit several thrombogenic characteristics such as neointimal hyperplasia, neoatherosclerosis and disrupted hemodynamics.^34,35^ This persistent thrombotic substrate likely makes them less tolerant to DAPT discontinuation, even after a stent-free intervention. A similar, non-significant, signal was seen in in the REC-CAGEFREE II trial.^13^ While this was an exploratory finding and our findings are subject to the same limitations as the rest of the analyses, the signal is biologically plausible, and its reproducibility might warrant further investigation in sufficiently powered trials.

Lastly, there was an absence of significant reduction of bleeding events among patients in the abbreviated treatment arm. While the findings were not statistically significant, there is no clear mechanistic explanation for the numerically increased cumulative incidence of bleeding events under abbreviated DAPT in the absence of treatment-assignment bias. Consistent with this, ATO analyses showed smaller between-group differences than the primary ATE model. This points to possible residual confounding from high bleeding risk patients being preferentially channelled into the abbreviated arm. With this said, the low statistical power across all secondary endpoints indicate that these findings should be interpreted with caution.

### Limitations

First, treatment allocation was not random. The design entails distinct risks for unmeasured confounding and misclassification of exposure. For instance, misclassification, likely primarily driven by non-adherence in 12-month arm, might be biasing our outcome measure towards the null and could increase the risk of Type II-error. Second, since our results rely on adjusted estimates, there is a risk of model-misspecification affecting results. Third, Shi *et al*.,^25^ amongst others, have shown that one cannot safely assume a class effect for DCBs. We partly mitigated this by excluding early DCB types known to be distinctly inferior, Supplementary material online, *Figure S1*. Fourth, the study is almost exclusively based on paclitaxel-coated balloons, which limits interpretability in the modern DCB era. Fifth, the exclusion of non-ticagrelor P2Y12 inhibitors decreases generalizability to other drugs and clinical settings but was done to reduce heterogeneity and explorative analyses of other P2Y12-inhibitors showed distinct positivity-violations. Sixth, use of ITT-data in ticagrelor patients likely has a higher risk of side-effect-driven non-adherence (e.g. dyspnoea) than other ITT variables and the degree of such an effect cannot be determined in our data. Seventh, due to data constraints we had to use index-admission-only ICD-codes to create the frailty-proxy variable.^31^ While key markers of frailty are likely still accurately represented (i.e. sequelae of stroke) the sensitivity of this variable in detecting all relevant aspects of frailty is decreased. Lastly, as shown by the Holm–Bonferroni-table (see Supplementary material online, *Table S10*) all secondary analyses should be regarded as exploratory.

## Conclusions

Our findings suggest that real-world ACS patients receiving DCB-only PCI differ meaningfully from those enrolled in the only randomized trial supporting abbreviated DAPT in this setting. Abbreviated DAPT was not associated with a statistically significant difference in NACE compared with Standard 12-month treatment. However, the confidence intervals were wide and did not exclude clinically meaningful harm. These findings should be regarded as hypothesis-generating and indicate the need for more comprehensive evidence before abbreviated DAPT is routinely adopted in this setting.

## Supplementary Material

pvag032_Supplementary_Data

## Data Availability

The data used in this study are held by the Swedish National Board of Health and Welfare and SWEDEHEART and cannot be shared publicly due to national legal restrictions. Upon publication of the manuscript, the R-code used for the primary analyses along with the statistical analysis plan will be made public.

## References

[1] Jeger RV, Eccleshall S, Wan Ahmad WA, Ge J, Poerner TC, Shin E-S, Alfonso F, Latib A, Ong PJ, Rissanen TT, Saucedo J, Scheller B, Kleber FX; International DCB Consensus Group. Drug-coated balloons for coronary artery disease. JACC Cardiovasc Interv 2020;13:1391–1402. 10.1016/j.jcin.2020.02.043 32473887

[2] Yerasi C, Case BC, Forrestal BJ, Torguson R, Weintraub WS, Garcia-Garcia HM, Waksman R. Drug-coated balloon for de novo coronary artery disease: JACC state-of-the-art review. J Am Coll Cardiol 2020;75:1061–1073. 10.1016/j.jacc.2019.12.046 32138967

[3] Jeger RV, Farah A, Ohlow M-A, Mangner N, Möbius-Winkler S, Leibundgut G, Weilenmann D, Wöhrle J, Richter S, Schreiber M, Mahfoud F, Linke A, Stephan FP, Mueller C, Rickenbacher P, Coslovsky M, Gilgen N, Osswald S, Kaiser C, Scheller B; BASKET-SMALL 2 Investigators. Drug-coated balloons for small coronary artery disease (BASKET-SMALL 2): an open-label randomised non-inferiority trial. Lancet 2018;392:849–856. 10.1016/S0140-6736(18)31719-7 30170854

[4] Korjian S, McCarthy KJ, Larnard EA, Cutlip DE, McEntegart MB, Kirtane AJ, Yeh RW. Drug-coated balloons in the management of coronary artery disease. Circ Cardiovasc Interv 2024;17:. 10.1161/circinterventions.123.013302 38771909

[5] Her A-Y, Ahmad WAW, Bang LH, Kiam OT, Nuruddin AA, Hsieh IC, Hwa HH, Yahaya SA, Tang Q, Hsu J-C, Qiu C, Qian J, Ali RM, Shin ES. Drug-coated balloon-based intervention for coronary artery disease: the second report of Asia-Pacific consensus group. JACC Asia 2025;5:701–717. 10.1016/j.jacasi.2025.02.017 PMC12287746 40304645

[6] Soleimani H, Karimi E, Mahalleh M, Entezari FJ, Nasrollahizadeh A, Nasrollahizadeh A, Rafiee H, Kalhor P, Al-Azizi KM, Rios LHP, Aronow WS, Ambrosy AP, Hosseini K. Abbreviated dual antiplatelet therapy in patients undergoing percutaneous coronary intervention: a systematic review and meta-analysis of randomized controlled trials. BMC Cardiovasc Disord 2025;25:343. 10.1186/s12872-025-04765-x 40307711 PMC12044780

[7] Valgimigli M, Frigoli E, Heg D, Tijssen J, Jüni P, Vranckx P, Ozaki Y, Morice M-C, Chevalier B, Onuma Y, Windecker S, Tonino PAL, Roffi M, Lesiak M, Mahfoud F, Bartunek J, Hildick-Smith D, Colombo A, Stanković G, Iñiguez A, Schultz C, Kornowski R, Ong PJL, Alasnag M, Rodriguez AE, Moschovitis A, Laanmets P, Donahue M, Leonardi S, Smits PC; MASTER DAPT Investigators. Dual antiplatelet therapy after PCI in patients at high bleeding risk. N Engl J Med 2021;385:1643–1655. 10.1056/nejmoa2108749 34449185

[8] Natsuaki M, Watanabe H, Morimoto T, Yamamoto K, Obayashi Y, Nishikawa R, Ando K, Domei T, Suwa S, Ogita M, Isawa T, Takenaka H, Yamamoto T, Ishikawa T, Hisauchi I, Wakabayashi K, Onishi Y, Hibi K, Kawai K, Yoshida R, Suzuki H, Nakazawa G, Kusuyama T, Morishima I, Ono K, Kimura T. An aspirin-free versus dual antiplatelet strategy for coronary stenting: STOPDAPT-3 randomized trial. Circulation 2024;149:585–600. 10.1161/circulationaha.123.066720 37994553

[9] Rao SV, O’Donoghue ML, Ruel M, Rab T, Tamis-Holland JE, Alexander JH, Baber U, Baker H, Cohen MG, Cruz-Ruiz M, Davis LL, de Lemos JA, DeWald TA, Elgendy IY, Feldman DN, Goyal A, Isiadinso I, Menon V, Morrow DA, Mukherjee D, Platz E, Promes SB, Sandner S, Sandoval Y, Schunder R, Shah B, Stopyra JP, Talbot AW, Taub PR, Williams MS. 2025 ACC/AHA/ACEP/NAEMSP/SCAI guideline for the management of patients with acute coronary syndromes: a report of the American College of Cardiology/American Heart Association joint committee on clinical practice guidelines. Circulation 2025;151:e771–e862. 10.1161/cir.0000000000001309 40014670

[10] Gorog DA, Ferreiro JL, Ahrens I, Ako J, Geisler T, Halvorsen S, Huber K, Jeong Y-H, Navarese EP, Rubboli A, Sibbing D, Siller-Matula JM, Storey RF, Tan JWC, Ten Berg JM, Valgimigli M, Vandenbriele C, Lip GYH. De-escalation or abbreviation of dual antiplatelet therapy in acute coronary syndromes and percutaneous coronary intervention: a consensus statement from an international expert panel on coronary thrombosis. Nat Rev Cardiol 2023;20:830–844. 10.1038/s41569-023-00901-2 37474795

[11] Byrne RA, Rossello X, Coughlan JJ, Barbato E, Berry C, Chieffo A, Claeys MJ, Dan G-A, Dweck MR, Galbraith M, Gilard M, Hinterbuchner L, Jankowska EA, Jüni P, Kimura T, Kunadian V, Leosdottir M, Lorusso R, Pedretti RFE, Rigopoulos AG, Rubini Gimenez M, Thiele H, Vranckx P, Wassmann S, Wenger NK, Ibanez B; ESC Scientific Document Group. 2023 ESC guidelines for the management of acute coronary syndromes. Eur Heart J 2023;44:3720–3826. 10.1093/eurheartj/ehad191 37622654

[12] Muramatsu T, Kozuma K, Tanabe K, Morino Y, Ako J, Nakamura S, Yamaji K, Kohsaka S, Amano T, Kobayashi Y, Ikari Y, Kadota K, Nakamura M; Task Force of the Japanese Association of Cardiovascular Intervention, Therapeutics (CVIT). Clinical expert consensus document on drug-coated balloon for coronary artery disease from the Japanese Association of Cardiovascular Intervention and Therapeutics. Cardiovasc Interv Ther 2023;38:166–176. 10.1007/s12928-023-00921-2 36847902 PMC10020262

[13] Gao C, Zhu B, Ouyang F, Wen S, Xu Y, Jia W, Yang P, He Y, Zhong Y, Zhou Y, Guo Z, Shen G, Ma L, Xu L, Xue Y, Hu T, Wang Q, Liu Y, Zhang R, Liu J, Jiang Z, Xia J, Garg S, van Geuns RJ, Capodanno D, Onuma Y, Wang D, Serruys P, Tao L; REC-CAGEFREE II Investigators. Stepwise dual antiplatelet therapy de-escalation in patients after drug coated balloon angioplasty (REC-CAGEFREE II): multicentre, randomised, open label, assessor blind, non-inferiority trial. BMJ 2025;388:e082945. 10.1136/bmj-2024-082945 40164448 PMC11955879

[14] Yu X, Wang X, Ji F, Zhang W, Yang C, Xu F, Wang F. A non-inferiority, randomized clinical trial comparing paclitaxel-coated balloon versus new-generation drug-eluting stents on angiographic outcomes for coronary de novo lesions. Cardiovasc Drugs Ther 2022;36:655–664. 10.1007/s10557-021-07172-4 33713211 PMC9270292

[15] Rissanen TT, Uskela S, Eränen J, Mäntylä P, Olli A, Romppanen H, Siljander A, Pietilä M, Minkkinen MJ, Tervo J, Kärkkäinen JM; DEBUT Trial Investigators. Drug-coated balloon for treatment of de-novo coronary artery lesions in patients with high bleeding risk (DEBUT): a single-blind, randomised, non-inferiority trial. Lancet 2019;394:230–239. 10.1016/S0140-6736(19)31126-2 31204115

[16] Vos NS, Fagel ND, Amoroso G, Herrman JR, Patterson MS, Piers LH, van der Schaaf RJ, Slagboom T, Vink MA. Paclitaxel-coated balloon angioplasty versus drug-eluting stent in acute myocardial infarction: the REVELATION randomized trial. JACC Cardiovasc Interv 2019;12:1691–1699. 10.1016/j.jcin.2019.04.016 31126887

[17] Takahashi T, Yamaji K, Kohsaka S, Ishii H, Mori Y, Kikuta Y, Wakatsuki T, Yamaguchi K, Nishioka D, Kusunose K, Amano T, Sata M, Kozuma K; J-PCI Registry Investigators. Successful or uncomplicated use of drug-coated balloon versus drug-eluting stent strategies for de novo culprit lesions in acute coronary syndromes: insights from a nationwide registry in Japan. J Am Heart Assoc 2025;14:e038071. 10.1161/jaha.124.038071 40439158 PMC12229198

[18] Mehran R, Rao SV, Bhatt DL, Gibson CM, Caixeta A, Eikelboom J, Kaul S, Wiviott SD, Menon V, Nikolsky E, Serebruany V, Valgimigli M, Vranckx P, Taggart D, Sabik JF, Cutlip DE, Krucoff MW, Ohman EM, Steg PG, White H. Standardized bleeding definitions for cardiovascular clinical trials. Circulation 2011;123:2736–2747. 10.1161/circulationaha.110.009449 21670242

[19] Skeppholm M, Friberg L. Usefulness of Health Registers for detection of bleeding events in outcome studies. Thromb Haemost 2016;116:1131–1139. 10.1160/th16-05-0400 27617328

[20] Thygesen K, Alpert JS, Jaffe AS, Chaitman BR, Bax JJ, Morrow DA, White HD. Fourth universal definition of myocardial infarction (2018). Circulation 2018;138:e618–e651. 10.1161/cir.0000000000000617 30571511

[21] Håkansson A, Koul S, Omerovic E, Andersson J, James S, Agewall S, Mokhtari A, van Der Pals J, Wester A, Szummer K, Jernberg T, Erlinge D, Mohammad MA. Abbreviated versus standard dual antiplatelet therapy times after percutaneous coronary intervention in patients with high bleeding risk with acute coronary syndrome: insights from the SWEDEHEART registry. J Am Heart Assoc 2024;13:e034709. 10.1161/JAHA.124.034709 38934886 PMC11255705

[22] Hernán MA, Robbins JM. Causal Inference: What If. Boca Raton: Chapman & Hall/CRC; 2025. 157–166; 221–244. https://miguelhernan.org/whatifbook

[23] Brookhart MA, Schneeweiss S, Rothman KJ, Glynn RJ, Avorn J, Stürmer T. Variable selection for propensity score models. Am J Epidemiol 2006;163:1149–1156. 10.1093/aje/kwj149 16624967 PMC1513192

[24] Urban P, Gregson J, Owen R, Mehran R, Windecker S, Valgimigli M, Varenne O, Krucoff M, Saito S, Baber U, Chevalier B, Capodanno D, Morice MC, Pocock S. Assessing the risks of bleeding vs thrombotic events in patients at high bleeding risk after coronary stent implantation. JAMA Cardiol 2021;6:410. 10.1001/jamacardio.2020.6814 33404627 PMC7788509

[25] Shi B, Wang HY, Liu J, Cai Z, Song C, Yin D, Wang H, Dong Q, Song W, Dou KF. Prognostic value of machine-learning-based PRAISE score for ischemic and bleeding events in patients with acute coronary syndrome undergoing percutaneous coronary intervention. J Am Heart Assoc 2023;12:e025812. 10.1161/JAHA.122.025812 36974761 PMC10122888

[26] Austin PC . An introduction to propensity score methods for reducing the effects of confounding in observational studies. Multivariate Behav Res 2011;46:399–424. 10.1080/00273171.2011.568786 21818162 PMC3144483

[27] Li F, Morgan KL, Zaslavsky AM. Balancing covariates via propensity score weighting. J Am Stat Assoc 2018;113:390–400. 10.1080/01621459.2016.1260466

[28] Conner SC, Sullivan LM, Benjamin EJ, LaValley MP, Galea S, Trinquart L. Adjusted restricted mean survival times in observational studies. Stat Med 2019;38:3832–3860. 10.1002/sim.8206 31119770 PMC7534830

[29] StataCorp . Statistical Software: Release 19. College Station, TX: StataCorp LLC.

[30] R Core Team . 2021. R: A language for environment for statistical computing. Vienna, Austria: R Foundation for Statistical Computing.

[31] Gilbert T, Neuburger J, Kraindler J, Keeble E, Smith P, Ariti C, Arora S, Street A, Parker S, Roberts HC, Bardsley M, Conroy S. Development and validation of a Hospital Frailty Risk Score focusing on older people in acute care settings using electronic hospital records: an observational study. Lancet 2018;391:1775–1782. 10.1016/s0140-6736(18)30668-8 29706364 PMC5946808

[32] Mathur MB, Ding P, Riddell CA, VanderWeele TJ. Web site and R package for computing E-values. Epidemiology 2018;29:e45–e47. 10.1097/ede.0000000000000864 29912013 PMC6066405

[33] Thrane PG, Olesen KKW, Thim T, Gyldenkerne C, Mortensen MB, Kristensen SD, Maeng M. Mortality trends after primary percutaneous coronary intervention for ST-segment elevation myocardial infarction. J Am Coll Cardiol 2023;82:999–1010. 10.1016/j.jacc.2023.06.025 37648359

[34] Yeh RW, Shlofmitz R, Moses J, Bachinsky W, Dohad S, Rudick S, Stoler R, Jefferson BK, Nicholson W, Altman J, Bateman C, Krishnaswamy A, Grantham JA, Zidar FJ, Marso SP, Tremmel JA, Grines C, Ahmed MI, Latib A, Tehrani B, Abbott JD, Batchelor W, Underwood P, Allocco DJ, Kirtane AJ; AGENT IDE Investigators. Paclitaxel-coated balloon vs uncoated balloon for coronary in-stent restenosis: the AGENT IDE randomized clinical trial. JAMA 2024;331:1015–1024. 10.1001/jama.2024.1361 38460161 PMC10924708

[35] Byrne RA, Joner M, Tada T, Kastrati A. Restenosis in bare metal and drug-eluting stents: distinct mechanistic insights from histopathology and optical intravascular imaging. Minerva Cardioangiol 2012;60:473–489. 23018428

