# Supplementary material for: Abbreviated dual antiplatelet therapy after percutaneous coronary intervention with drug coated balloons in acute coronary syndromes: insights from the SWEDEHEART registry
Source: Eur Heart J Cardiovasc Pharmacother. 2026 May 7;12(4):238–46. doi: 10.1093/ehjcvp/pvag032 (PMC13367248; doi:10.1093/ehjcvp/pvag032)
Supplement: pvag032_Supplementary_Data [file pvag032_supplementary_data.docx]

Supplemental material:
Abbreviated versus standard DAPT duration after PCI with drug coated balloons in acute coronary syndromes.

Anton Håkansson MD^1^, Sacharias von Koch MD^1^, Axel Dahlgren MD^1^, Christian Reitan MD, PhD^2^, Sasha Koul MD, PhD^1^, Prof Stefan James, MD, PhD ^3^, Prof Tomas Jernberg MD, PhD^4^, Per Grimfjärd MD, PhD^5^, Prof Elmir Omerovic MD, PhD^6^, Oskar Angerås MD, PhD^7^ Prof David Erlinge MD, PhD ^1^, Moman A. Mohammad MD, PhD^1^

1) Division of Cardiology, Department of Clinical Sciences Lund, Lund University, Lund, Sweden

2) Department of Clinical Sciences, Cardiology, Karolinska Institutet, Danderyd Hospital, Stockholm, Sweden.

3) Dept. of Medical Sciences Uppsala University, Uppsala Sweden

4) Department of clinical sciences, Danderyd hospital, Karolinska Institutet, Stockholm, Sweden.

5) Västerås Hospital, Västerås, Sweden; Dept of Medical Sciences

6) Division of Cardiology, Department of Molecular and Clinical Medicine, Sahlgrenska Academy, University of Gothenburg, Gothenburg, Sweden.

7) Department of Thoracic surgery and Cardiology, Sahlgrenska Univseristy Hospital, Gothenburg, Sweden and Insitute of Medicine, Sahlgrenska Academy, Univeristy of Gothenburg, Sweden.

**Correspondence to:** Anton Håkansson, Department of Cardiology, Clinical Sciences, Lund University, Skane University Hospital, Lund; 221 85 Lund, Sweden; telephone: +46 709 799 478; email: anton.hakansson@med.lu.se

Table of contents

[Supplemental material: Abbreviated versus standard DAPT duration after PCI with drug coated balloons in acute coronary syndromes. 1](#_Toc227791526)

[**Supplementary Table 1.** Definitions and variables 3](#_Toc227791527)

[**Supplementary Table 2.** Variables included in IPTW models 4](#_Toc227791528)

[**Supplementary Table 3.** PCI and lesion data 5](#_Toc227791529)

[Supplementary Table 4. IPTW diagnostics 7](#_Toc227791530)

[Supplementary table 5. Details on the calculated frailty-proxy variable used in IPTW 8](#_Toc227791531)

[**Supplementary Table 6.** Table of incidence rates for primary and secondary endpoints 10](#_Toc227791532)

[**Supplementary Table 7.** Frequency of different bleeding events 11](#_Toc227791533)

[**Supplementary Table 8.** Sensitivity analyses 12](#_Toc227791534)

[**Supplementary Table 9.** Results of different IPTW adjustment models 13](#_Toc227791535)

[**Supplementary table 10.** Holm-Bonferroni gated analysis plan 14](#_Toc227791536)

[**Supplementary Figure 1. Distribution of probability of treatment** 15](#_Toc227791537)

[Supplementary Figure 2. 16](#_Toc227791538)

[**Supplementary Figure 3. Test of proportional hazards** 17](#_Toc227791539)

## **Supplementary Table 1.** Definitions and variables

|  | **Source** | **Definition** |
| --- | --- | --- |
| **Net adverse clinical events** | National patient registry  National population registry  SWEDEHEART | All-cause death, myocardial infarction, ischemic stroke or bleeding. |
| **Major bleeding** | National patient registry | Extracted to match BARC 2&3 events. In-patient registry of one or more of the following diagnoses:  **Hemorrhagic stroke:**  ICD–10: I60–I62.  **Gastrointestinal bleeding:**  ICD–10: K226, K250, K252, K254, K256, K260, K262, K264, K266, K270, K272, K274, K276, K280, K282, K284, K286, K290, K625, K920, K921, K922, I850.  **Anemia–related bleeding:**  ICD–10: D629, D500.  **Other bleeding:**  ICD–10: N421, N938, N939, N950, R041, R042, R048, R049, R210, R319, R210, T810, N501A |
|  |  |  |
| **Myocardial infarction** | SWEDEHEART registry | New registration for MI in the SWEDEHEART registry with a discharge diagnosis of MI according to the fourth universal definition of MI  ICD–10: I21–I22. |
| **Ischemic stroke** | National patient registry | ICD–10: I63.0–I63.6. |
| **All cause death** | National population registry | All recorded deaths in the registry.  Includes death caused by bleeding. |
| **Variables in multivariable regression** | SWEDEHEART registry | 1) Age 2) eGFR 3) Major bleeding within 1 year of admission 4) Type of ACS 5) De novo or in-stent lesion. |
| **Inverse probability of treatment weighting** | R-packages used:  "haven", "dplyr", "tidyr", "survival", "survminer", "WeightIt", "cobalt", "sandwich", "broom", "ggplot2", "scales", "survey", "survRM2", "tibble", "patchwork", "ggplotify", "gtable", "purrr" | Primary estimand: Average Treatment Effect (ATE) Also reported: Average treatment on overlap weights (ATO)  Untrimmed and trimmed weights (1 and 99^th^ percentil) were used.  Three different weighting models were fitted. |
| **Augumented inverse probability of treatment** | R-packages used: "AIPW", "SuperLearner", "glmnet", "ranger", "dplyr", "tibble" | SL.glm, SL.glm.interaction, SL.step.interaction.  5-fold crossfitting.  g-bound (default in package): 0.025 |
| **Restricted mean survival time** | R-packages used: library(WeightIt)  library(survival)  library(dplyr)  library(ggplot2) | To estimate standard errors and 95% CI in the IPTW population, bootstrapping was used, refitting the the PS model in each replicate. |

**Table S1.** Definitions of primary and secondary outcomes in survival analyses as well as variables used for sensitivity analysis of primary composite outcome (net adverse clinical events).

*MI=myocardial infarction, STEMI=ST-elevation myocardial infarction, NSTEMI=non-ST-elevation myocardial infarction, UA=Unstable angina pectoris, SWEDEHEART=* *Swedish Web-System for Enhancement and Development of Evidence-Based Care in Heart Disease Evaluated According to Recommended Therapies, BARC=Bleeding academic research consortium, eGFR=estimated glomerular filtration rate, ACS=Acute coronary syndrome, SL=superlearner, IPTW=Inverse probability of treatment weight, PS=Propensity score, CI=Confidence interval.*

## **Supplementary Table 2.** Variables included in IPTW models

| **Primary model** | **Slim model** | **Extensive model** |
| --- | --- | --- |
| Age  Smoking status  Diabetes  Hypertension  Previous MI  Admission diagnosis of CHF  Admission diagnosis of CKD  Previous bleeding (1 year)  Previous stroke  Admission diagnosis of cancer  Anemia (<11g/l)  Type of ACS  Nr of coronary vessels with disease  De novo or in-stent lesion  Frailty proxy-variable | Age  eGFR  Previous bleeding (1 year)  Type of ACS  De novo or in-stent lesion | Age  Gender  Smoking status  Site of punction  ACEi at discharge  ARB at discharge  Betablocker at discharge  Statin at discharge  Calciummodification  Diabetes  Hypertension  Previous MI  Nr of lesions treated  Previous PCI  Previous CABG  Admission diagnosis of CHF  Admission diagnosis of peripheral artery disease  Admission diagnosis of COPD  Admission diagnosis of CKD  Previous bleeding (1 year)  Previous stroke  Admission diagnosis of cancer  Anemia (<11g/l)  Type of ACS  Nr of coronary vessels with disease  Type of lesion (i.e bifurcation)  De novo or in-stent lesion  Frailty proxy-variable |
| **Screened but not included variables** | 1. Year of PCI (instrument) 2. Centre id (instrument) 3. LVEF (too many missing) 4. Peak troponing (too many missing) |  |

**Table S2.** Table showing which variable was used for each IPTW model. Red variables are used in the slim model. Green variables are used only in the extensive model.

## **Supplementary Table 3.** PCI and lesion data

| **Characteristic** | **Overall**  N = 1,128^1^ | **Standard DAPT**  N = 987^1^ | **Abbreviated DAPT**  N = 141^1^ |
| --- | --- | --- | --- |
| **Treated segment** |  |  |  |
| Proximal RCA | 65 (5.8%) | 55 (5.6%) | 10 (7.1%) |
| Mid RCA | 83 (7.4%) | 72 (7.3%) | 11 (7.8%) |
| Distal RCA | 41 (3.6%) | 38 (3.9%) | 3 (2.1%) |
| PDA/RPD | 35 (3.1%) | 32 (3.2%) | 3 (2.1%) |
| Left main | 22 (2.0%) | 21 (2.1%) | 1 (0.7%) |
| Proximal LAD | 139 (12.3%) | 120 (12.2%) | 19 (13.5%) |
| Mid LAD | 130 (11.5%) | 113 (11.4%) | 17 (12.1%) |
| Distal LAD | 43 (3.8%) | 37 (3.7%) | 6 (4.3%) |
| First diagonal | 175 (15.5%) | 155 (15.7%) | 20 (14.2%) |
| Second diagonal | 53 (4.7%) | 46 (4.7%) | 7 (5.0%) |
| Proximal LCx | 76 (6.7%) | 65 (6.6%) | 11 (7.8%) |
| First obtuse marginal | 99 (8.8%) | 89 (9.0%) | 10 (7.1%) |
| Second obtuse marginal | 35 (3.1%) | 32 (3.2%) | 3 (2.1%) |
| Distal LCx | 51 (4.5%) | 41 (4.2%) | 10 (7.1%) |
| LPD | 10 (0.9%) | 9 (0.9%) | 1 (0.7%) |
| Posterolateral from left | 3 (0.3%) | 3 (0.3%) | 0 (0.0%) |
| Intermediate | 41 (3.6%) | 37 (3.7%) | 4 (2.8%) |
| Posterolateral | 20 (1.8%) | 15 (1.5%) | 5 (3.5%) |
| Right ventricular branch | 0 (0.0%) | 0 (0.0%) | 0 (0.0%) |
| Septal | 7 (0.6%) | 7 (0.7%) | 0 (0.0%) |
| **Angiographic finding** |  |  |  |
| Non-conclusive | 0 (0.0%) | 0 (0.0%) | 0 (0.0%) |
| Normal/atheroma | 0 (0.0%) | 0 (0.0%) | 0 (0.0%) |
| 1-vessel (non-LMCA) | 901 (79.9%) | 785 (79.5%) | 116 (82.3%) |
| 2-vessel (non-LMCA) | 155 (13.7%) | 139 (14.1%) | 16 (11.3%) |
| 3-vessel (non-LMCA) | 41 (3.6%) | 35 (3.5%) | 6 (4.3%) |
| LMCA + 1-vessel | 3 (0.3%) | 2 (0.2%) | 1 (0.7%) |
| LMCA + 2-vessel | 11 (1.0%) | 11 (1.1%) | 0 (0.0%) |
| LMCA + 3-vessel | 10 (0.9%) | 9 (0.9%) | 1 (0.7%) |
| LMCA | 7 (0.6%) | 6 (0.6%) | 1 (0.7%) |
| **Lesion type (modified ACC/AHA)** |  |  |  |
| A | 124 (11.0%) | 109 (11.0%) | 15 (10.6%) |
| B1 | 367 (32.5%) | 318 (32.2%) | 49 (34.8%) |
| B2 | 324 (28.7%) | 288 (29.2%) | 36 (25.5%) |
| C | 111 (9.8%) | 95 (9.6%) | 16 (11.3%) |
| B1 bifurcation | 115 (10.2%) | 98 (9.9%) | 17 (12.1%) |
| B2 bifurcation | 67 (5.9%) | 61 (6.2%) | 6 (4.3%) |
| C bifurcation | 20 (1.8%) | 18 (1.8%) | 2 (1.4%) |
| **Mean DCB diameter, mm*** | 2.7 (0.6) | 2.7 (0.6) | 2.8 (0.6) |
| **Mean DCB length, mm*** | 20.7 (5.4) | 20.9 (5.5) | 19.8 (5.0) |
| **DCB device** |  |  |  |
| Braun SeQuent Please | 615 (59.5%) | 539 (59.6%) | 76 (58.9%) |
| Ivatec In.Pact Falcon | 206 (19.9%) | 178 (19.7%) | 28 (21.7%) |
| Biotronik Pantera Lux | 162 (15.7%) | 141 (15.6%) | 21 (16.3%) |
| Boston Scientific Agent | 49 (4.7%) | 45 (5.0%) | 4 (3.1%) |
| Magictouch | 1 (0.1%) | 1 (0.1%) | 0 (0.0%) |
| **IVUS used** | 26 (100.0%) | 25 (100.0%) | 1 (100.0%) |
| **OCT used** | 39 (3.5%) | 35 (3.5%) | 4 (2.9%) |
| **Intracoronary pressure measurement** | 180 (16.0%) | 153 (15.5%) | 27 (19.3%) |
| **Any calcium modification** | 30 (2.7%) | 29 (2.9%) | 1 (0.7%) |
| **Access site** |  |  |  |
| Radial/ulnar | 927 (82.2%) | 812 (82.3%) | 115 (81.6%) |
| Femoral/large vessel | 201 (17.8%) | 175 (17.7%) | 26 (18.4%) |

**Table S3.** Description of detailed angiographic and procedural characteristics from index-PCI *PCI=Percutaneous coronary intervention, DAPT=Dual antiplatelet therapy, RCA=Right coronary artery, LAD=Left anterior decending artery, LCx=Left circumflex artery, LPD=Left posterior descending, LMCA=Left main coronary artery, DCB=Drug coated balloon, IVUS=Intravascular ultrasound, OCT=Optical coherence tomography.
** *For estimation of the mean DCB diameter and length in patients who were treated with multiple devices, the mean of all of the used devices was used per patient.*

## Supplementary Table 4. IPTW diagnostics

| Metric | Value |
| --- | --- |
| N (total) | 1127 |
| N abbreviated DAPT | 141 |
| N standard DAPT | 986 |
| Marginal P(treated) | 0.125 |
| — Stabilized ATE weights — |  |
| Mean (SD) | 1.00 (0.22) |
| Median [IQR] | 0.98 [0.94–1.03] |
| Min / Max | 0.281 / 3.53 |
| 99th percentile | 1.68 |
| Coefficient of variation | 0.22 |
| N weights > 10 | 0 |
| N weights > 20 | 0 |
| ESS, treated | 106 |
| ESS, control | 979 |
| ESS, total | 1076 |
| ESS retention, treated (%) | 75.4 |
| ESS retention, control (%) | 99.3 |
| — Unstabilized weights (reference) — |  |
| Median [IQR] | 1.14 [1.09–1.22] |
| Max | 28.22 |
| — Covariate balance (absolute SMD) — |  |
| Max \|SMD\|, unweighted | 0.131 |
| Max \|SMD\|, weighted | 0.057 |
| Mean \|SMD\|, unweighted | 0.042 |
| Mean \|SMD\|, weighted | 0.011 |
| N covariates with \|SMD\| > 0.10, unweighted | 3 |
| N covariates with \|SMD\| > 0.10, weighted | 0 |
| — Propensity score overlap — |  |
| PS range, treated | 0.035–0.445 |
| PS range, control | 0.017–0.549 |
| Common support region | 0.035–0.445 |
| N outside common support | 52 |

**Table S4.** Table with detailed description of the model diagnostics related to the primary IPTW model. *IPTW=Inverse probability of treatment weight, DAPT=Dual antiplatelet therapy, ATE=average treatment effect, ESS=Effective sample size, SMD=Standardized mean difference, PS=propensity score*

## Supplementary table 5. Details on the calculated frailty-proxy variable used in IPTW

| **ICD-10** | **Diagnosis** | **HFRS weight** | **n** | **% of cohort** | **Total HFRS points** |
| --- | --- | --- | --- | --- | --- |
| N18 | Chronic kidney disease | 1.4 | 63 | 5.59 | 88.2 |
| M48 | Other spondylopathies (incl. spinal stenosis) | 0.5 | 25 | 2.22 | 12.5 |
| N17 | Acute kidney failure | 1.8 | 24 | 2.13 | 43.2 |
| D64 | Other anaemias | 0.4 | 23 | 2.04 | 9.2 |
| I63 | Cerebral infarction (ischaemic stroke) | 0.8 | 19 | 1.68 | 15.2 |
| J96 | Respiratory failure NEC | 1.5 | 18 | 1.60 | 27.0 |
| I69 | Sequelae of cerebrovascular disease | 3.7 | 17 | 1.51 | 62.9 |
| M79 | Other soft tissue disorders | 1.1 | 17 | 1.51 | 18.7 |
| S72 | Fracture of femur | 1.4 | 15 | 1.33 | 21.0 |
| J18 | Pneumonia, organism unspecified | 1.1 | 13 | 1.15 | 14.3 |
| F32 | Depressive episode | 0.5 | 13 | 1.15 | 6.5 |
| K92 | Other diseases of digestive system (incl. GI bleed/melaena) | 0.8 | 12 | 1.06 | 9.6 |
| R31 | Unspecified haematuria | 3.0 | 11 | 0.98 | 33.0 |
| R00 | Abnormalities of heart beat | 0.7 | 11 | 0.98 | 7.7 |
| N19 | Unspecified kidney failure | 1.6 | 10 | 0.89 | 16.0 |
| Z99 | Dependence on enabling machines/devices NEC | 0.8 | 10 | 0.89 | 8.0 |
| K59 | Other functional intestinal disorders (incl. constipation) | 1.8 | 9 | 0.80 | 16.2 |
| F10 | Mental/behavioural disorders due to alcohol | 0.7 | 8 | 0.71 | 5.6 |
| I95 | Hypotension | 1.6 | 6 | 0.53 | 9.6 |
| M81 | Osteoporosis without pathological fracture | 1.4 | 6 | 0.53 | 8.4 |
| N20 | Calculus of kidney/ureter | 0.7 | 6 | 0.53 | 4.2 |
| G30 | Alzheimer’s disease | 4.0 | 4 | 0.35 | 16.0 |
| N28 | Other disorders of kidney and ureter | 1.3 | 3 | 0.27 | 3.9 |
| K52 | Other noninfective gastroenteritis/colitis | 0.3 | 3 | 0.27 | 0.9 |
| I67 | Other cerebrovascular diseases | 2.6 | 1 | 0.09 | 2.6 |
| K26 | Duodenal ulcer | 1.6 | 1 | 0.09 | 1.6 |
| M19 | Other arthrosis | 1.5 | 1 | 0.09 | 1.5 |
| J69 | Pneumonitis due to solids and liquids (aspiration) | 1.0 | 1 | 0.09 | 1.0 |
| M41 | Scoliosis | 0.9 | 1 | 0.09 | 0.9 |
| M80 | Osteoporosis with pathological fracture | 0.8 | 1 | 0.09 | 0.8 |
| J22 | Unspecified acute lower respiratory infection | 0.7 | 1 | 0.09 | 0.7 |
| M15 | Polyarthrosis | 0.4 | 1 | 0.09 | 0.4 |
| *Codes recorded in zero patients (n = 77):* F00, G81, R29, F05, S00, N39, W19, B96, R41, R26, R56, R40, S06, T83, M25, E86, E87, S42, R54, W18, Z50, F03, F01, Z75, S80, L03, E53, H54, Z60, G20, R55, S22, L89, Z22, B95, R44, L97, A41, Z87, X59, G40, R94, E16, S32, R33, R69, G31, Y95, R45, S09, G45, R32, A09, Z74, S01, A04, W06, Z93, E55, R02, R47, W01, W10, E05, R63, H91, R13, U80, Y84, R79, Z73, Z91, S51, E83, L08, R11, R50 | | | | | |

**Table S5.** The HFRS is calculated from a fixed list of 109 ICD-10 codes, each carrying a pre-specified weight. In this study, codes were ascertained from diagnoses recorded at the index admission only, rather than the two-year look-back window used in the original Gilbert et al. specification. Each code counts once per patient regardless of how many times it appears in the record. A patient's total HFRS score is the sum of the weights of all codes they had. The table shows the 32 codes recorded in at least one patient, ranked by how common each was in the cohort. "n" is the number of patients with the code, "% of cohort" is that number as a percentage of 1,127, and "Total HFRS points" is the weight multiplied by n, i.e. how much that code contributed to the cohort's combined score. The 77 codes that were never recorded are listed together in the bottom row and contributed nothing to any patient's score. Across all recorded codes, *ACS, acute coronary syndrome; DCB, drug-coated balloon; HFRS, Hospital Frailty Risk Score; ICD-10, International Classification of Diseases, 10th revision; NEC, not elsewhere classified; PCI, percutaneous coronary intervention.*

## **Supplementary Table 6.** Table of incidence rates for primary and secondary endpoints

| Outcome | Crude % | | IPTW-weighted % | | Aalen–Johansen % (competing risk = death) | | N (abb / std) |
| --- | --- | --- | --- | --- | --- | --- | --- |
|  | **Abbreviated** | **Standard** | **Abbreviated** | **Standard** | **Abbreviated** | **Standard** |  |
| NACE | 17.7 | 13.5 | 17.8 | 13.8 | — | — | 141 / 986 |
| MI | 12.1 | 9.0 | 11.4 | 9.1 | 11.3 | 9.0 | 141 / 986 |
| All-cause death | 4.3 | 2.0 | 3.1 | 2.2 | — | — | 141 / 986 |
| Stroke | 0.7 | 0.6 | 0.8 | 0.7 | 0.7 | 0.7 | 141 / 986 |
| Major bleeding | 5.8 | 4.2 | 6.2 | 4.3 | 6.1 | 4.3 | 141 / 986 |

**Table S6.** Showing different estimands of the cumulative incidence rates for primary and secondary endpoint. Crude %: unadjusted cumulative incidence at 365 days (events/N). IPTW-weighted %: cumulative incidence after stabilized ATE weighting. Aalen–Johansen %: cumulative incidence function accounting for the competing risk of death; reported for non-fatal components only. NACE and all-cause death are not reported under Aalen–Johansen because NACE includes death as a component and all-cause death has no competing risk.
*NACE=Net adverse clinical events, IPTW=inverse probability of treatment weight, MI=myocardial infarction.*

## **Supplementary Table 7.** Frequency of different bleeding events

| T810 | 25 | Haemorrhage and haematoma complicating a procedure |
| --- | --- | --- |
| K922 | 24 | Gastrointestinal haemorrhage, unspecified |
| R319 | 21 | Haematuria, unspecified |
| K921 | 13 | Melaena |
| D629 | 12 | Acute posthemorrhagic anaemia |
| K625 | 5 | Haemorrhage of anus and rectum |
| K260 | 4 | Duodenal ulcer, acute with haemorrhage |
| K920 | 4 | Haematemesis |
| R042 | 4 | Haemoptysis |
| I619 | 3 | Intracerebral haemorrhage, unspecified |
| K250 | 2 | Gastric ulcer, acute with haemorrhage |
| D500 | 1 | Iron deficiency anaemia secondary to blood loss (chronic) |
| I610 | 1 | Intracerebral haemorrhage, hemispheric, subcortical |
| I618 | 1 | Other intracerebral haemorrhage |
| I620 | 1 | Subdural haemorrhage (nontraumatic) |
| K254 | 1 | Gastric ulcer, chronic or unspecified with haemorrhage |

**Table S7** Frequency of all reported ICD-codes used for defining bleeding endpoint in secondary analysis. Frequency corresponds to the number of rows with respective ICD-code and are related to hospital stay only. One hospitalization can contribute with more than one ICD-code, causing grand sum of ICD-codes to be greater than number of bleeding events.

## **Supplementary Table 8.** Sensitivity analyses

| *Panel A. Alternative weighting strategies and Cox outcome regression* | | | |
| --- | --- | --- | --- |
| Outcome | **Trimmed IPTW HR (95% CI)** | **Overlap weighting HR (95% CI)** | **Multivariate Cox HR (95% CI); p** |
| NACE | 1.34 (0.86–2.09) | 1.17 (0.75–1.80) | 1.26 (0.82–1.95); 0.29 |
| All-cause death | 1.54 (0.60–3.97) | 1.39 (0.53–3.63) | 1.68 (0.65–4.37); 0.28 |
| Myocardial infarction | 1.31 (0.76–2.24) | 1.24 (0.73–2.10) | 1.32 (0.78–2.24); 0.31 |
| Stroke | 1.18 (0.14–9.76) | — | 0.87 (0.10–7.41); 0.90 |
| Major bleeding | 1.47 (0.64–3.35) | 1.20 (0.55–2.61) | 1.24 (0.57–2.69); 0.59 |
|  |  |  |  |
| *Panel B. Alternative estimands for the primary endpoint (NACE)* | | | |
| Method | **Estimand** | **Estimate (95% CI)** | **p-value** |
| AIPW | Risk difference, % | +5.6 (−2.9 to 14.1) | 0.20 |
| AIPW | Risk ratio | 1.40 (0.90–2.20) | 0.14 |
| IPTW-weighted RMST (τ=365 d) | Δ RMST, days | +0.72 (−23.34 to 19.31) | 0.87 |

**Table S8.** Trimmed IPTW: propensity-score trimming at the 1st/99th percentiles. Overlap weighting (ATO): weights proportional to probability of the opposite treatment; not estimable for stroke due to sparse events. Multivariate Cox: directly adjusted Cox model including age, eGFR, prior bleeding, and ACS presentation type. AIPW: doubly-robust augmented IPW with SuperLearner (SL.glm, SL.glm.interaction, SL.step.interaction) and 5-fold cross-fitting; 365-day binary outcome. RMST: IPTW-weighted restricted mean survival time at τ=365 days, calculated as abbreviated minus standard. RMST confidence intervals calculated using bootstraping.

## **Supplementary Table 9.** Results of different IPTW adjustment models

| Outcome | Events (total) | Abbreviated DAPT % (n/N) | Standard DAPT % (n/N) | Extensive IPTW* HR (95% CI), p | Slim IPTW† HR (95% CI), p |
| --- | --- | --- | --- | --- | --- |
| NACE | 158 | 17.7 (25/141) | 13.5 (133/986) | 1.34 (0.84–2.13), p=0.223 | 1.32 (0.83–2.08), p=0.238 |
| MI | 105 | 12.1 (17/141) | 9.0 (88/986) | 1.27 (0.72–2.22), p=0.406 | 1.36 (0.79–2.36), p=0.270 |
| All-cause death | 26 | 4.3 (6/141) | 2.0 (20/986) | 1.54 (0.58–4.13), p=0.389 | 2.36 (0.88–6.32), p=0.088 |
| Stroke | 7 | 0.7 (1/141) | 0.6 (6/986) | 1.55 (0.19–12.84), p=0.684 | 0.71 (0.09–5.89), p=0.749 |
| Major bleeding | 49 | 5.8 (8/141) | 4.2 (41/986) | 1.47 (0.61–3.54), p=0.390 | 1.32 (0.56–3.08), p=0.523 |

| **Table S9.** Table of the results of the alternative IPTW models, described in Table S2, for the primary endpoint along with secondary endpoint. Follow-up truncated at 365 days. Crude incidence rates reported as % (events / N) at 365 days.  * Extensive model: 41-covariate logistic propensity score; IPTW-weighted Cox with robust SE. |
| --- |
| † Slim model: reduced 5-variable covariate set; IPTW-weighted Cox with robust SE. |

## **Supplementary table 10.** Holm-Bonferroni gated analysis plan

| Family | Contents | α‑rule |
| --- | --- | --- |
| F1 (primary) | NACE composite - whole cohort  (adjusted p-value) | Single test @ α = 0.05 |
| F2 (key secondary) | 4 components (death, stroke, MI and major bleeding) | Holm on 4 tests, gated by significant F1 |
| F3 (subgroup) | NACE in in-stent-restenosis and in de novo lesions including interaction analysis | Exploratory |
| F4 | Primary endpoint evaluated with AIPW and RMST analysis. | Sensitivity analyses |

**Table S11.** Table displaying the Holm-Bonferroni gated analysis plan created post hoc by statistician without insight in data. Family groups indicate order of analysis, contents which type of analysis and alfa-rule indicates interpretation and gating to further analyses. All analyses are evaluated by result of the multivariable adjusted p-value (not crude) as recommended by literature.
*NACE=net adverse clinical events, MI=Myocardial infarction, IPTW=inverse probability of treatment weight. AIPW=Augmented inverse probability weight, RMST=Restricted Mean Survival Time.*

## **Supplementary Figure 1. Distribution of probability of treatment**


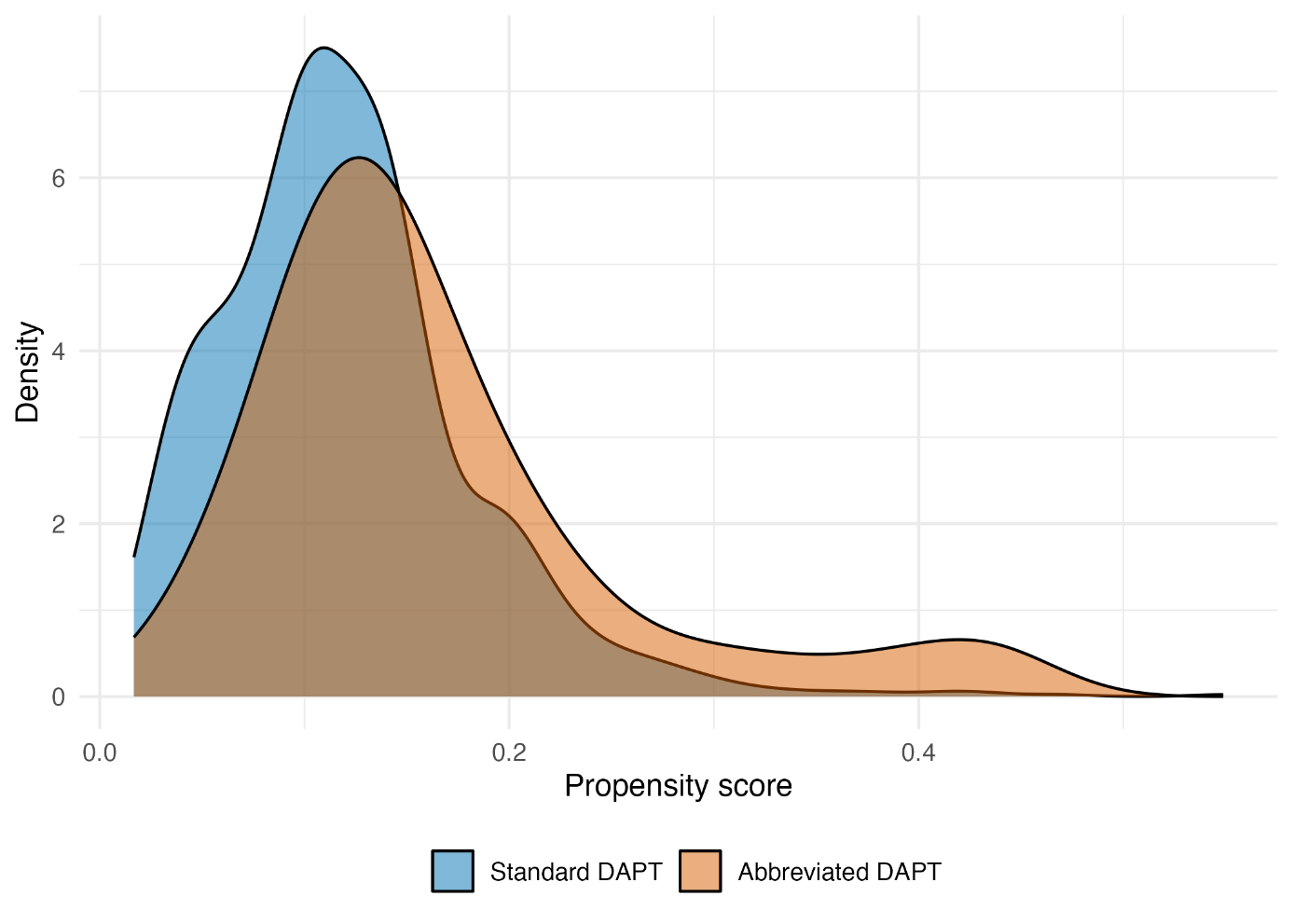


**Figure S1.** Density plot depicting the relative distribution density of the estimated propensity of receiving abbreviated DAPT based on covariate distribution in the primary inverse probability of treatment model.

Supplementary Figure 2.


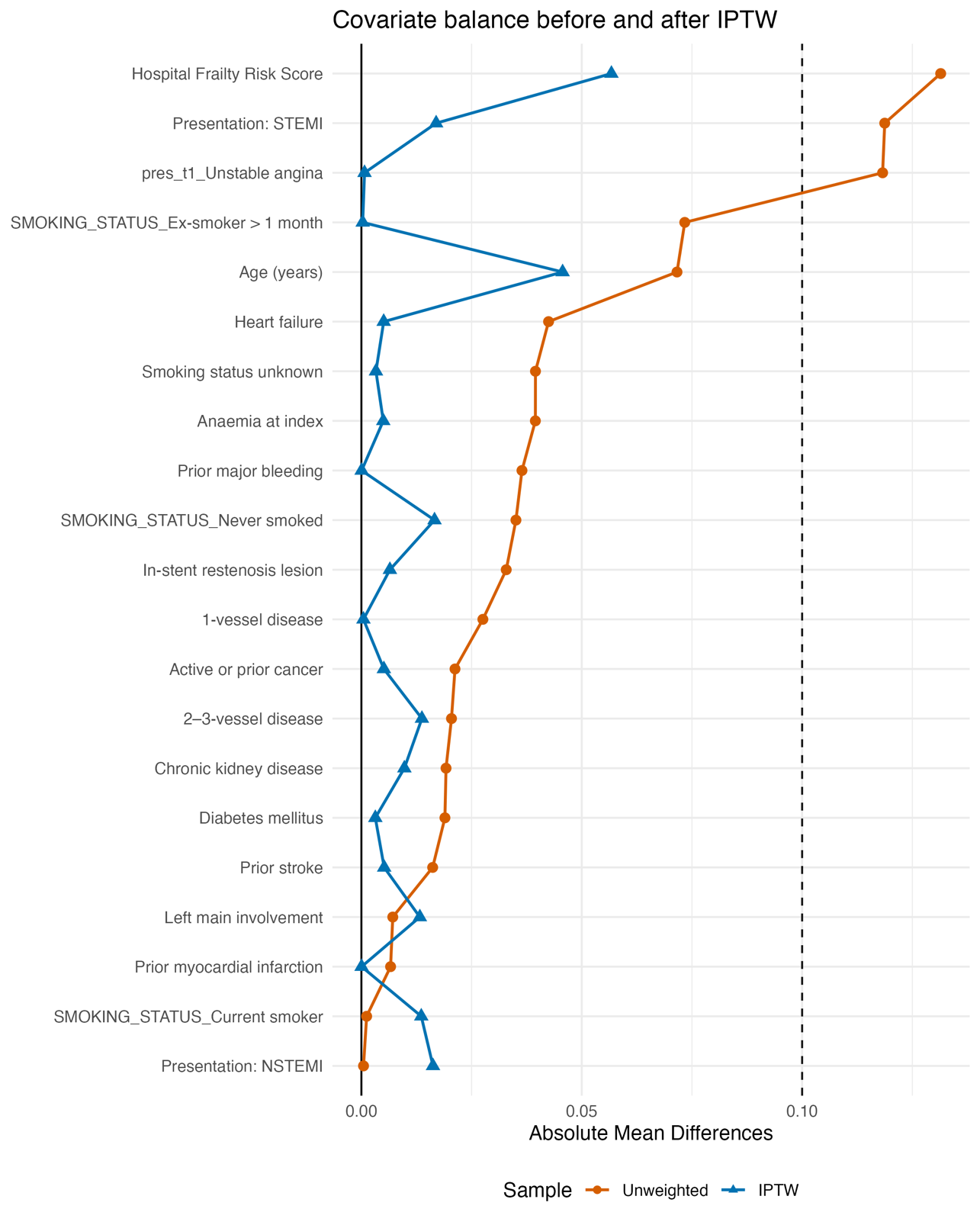


**Figure S2** Depicting the standardized mean differences among adjustment covariates between study groups before and after inverse probability of treatment re-weighting (IPTW). Results represent the results of the primary adjustment model.

## **Supplementary Figure 3. Test of proportional hazards**


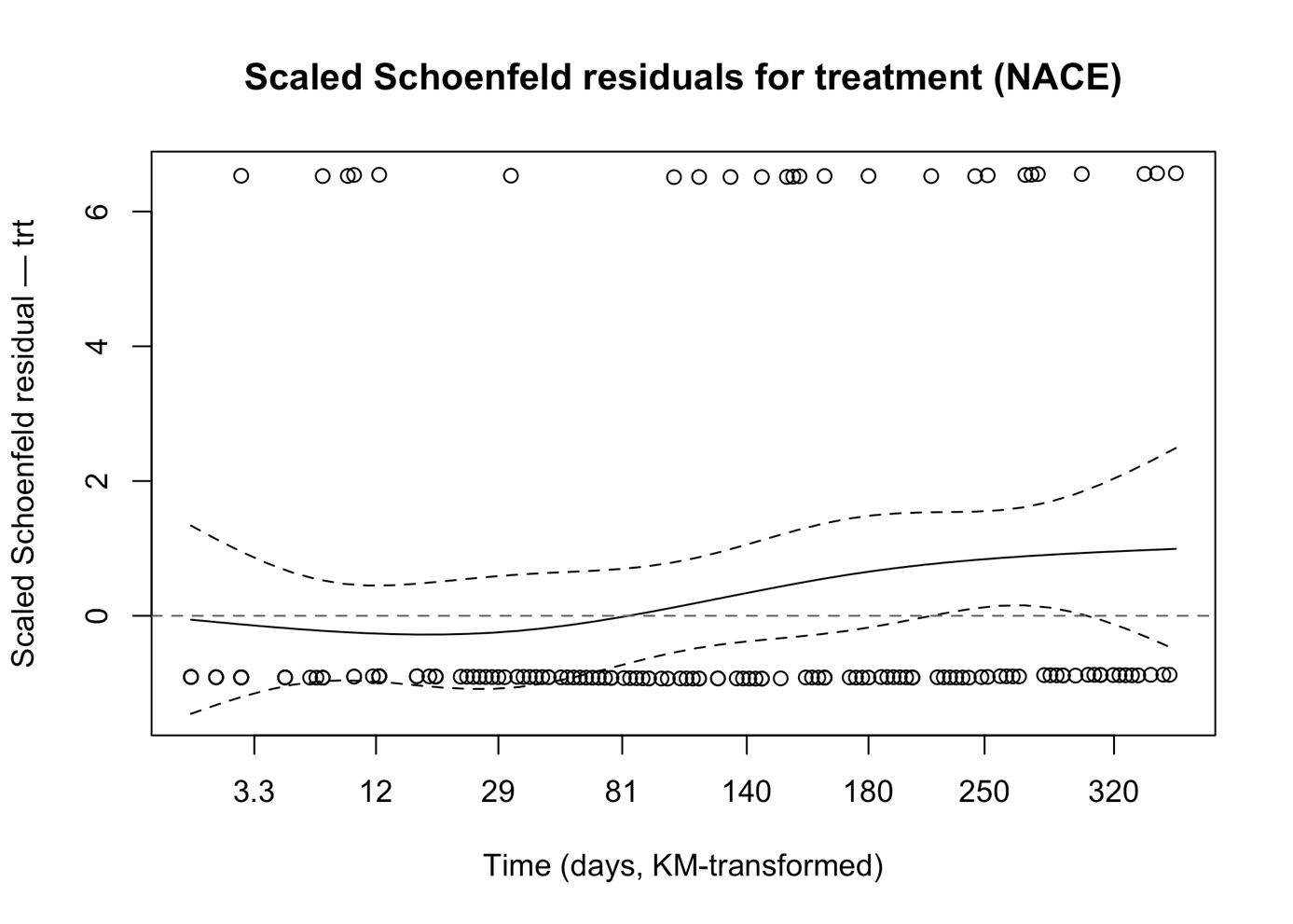


**Figure S3.** Figure representing the scaled shoenfeld residuals for treatment effect on the primary endpoint, net adverse clinical events, NACE. Empty circles represent NACE event among treatment arm (upper part of picture) and controll arm (lower part of picture). Whole line represents point estimate and interrupted line represent upper and lower confidence interval. Horizontal interrupted line represents completet proportionality of hazards throughout follow-up. The KM transform compresses calendar regions with few events and expands regions with many, so each residual occupies roughly equal horizontal space.
